# Data-driven profiles of attention-deficit/hyperactivity disorder using objective and ecological measures of attention, distractibility, and hyperactivity

**DOI:** 10.1007/s00787-023-02250-4

**Published:** 2023-06-30

**Authors:** Pilar Fernández-Martín, Rocío Rodríguez-Herrera, Rosa Cánovas, Unai Díaz-Orueta, Alma Martínez de Salazar, Pilar Flores

**Affiliations:** 1 Department of Psychology, Faculty of Psychology, University of Almeria, Carretera de Sacramento S/N, La Cañada de San Urbano, 04120 Almería, Spain; 2 Health Research Center (CEINSA), University of Almeria, Almería, Spain; 3 Neurorehabilitation and Autonomy Center Imparables, Almería, Spain; 4 Department of Psychology, Maynooth University, Maynooth, Ireland; 5 International University of La Rioja (UNIR), Logroño, Spain; 6 Child and Adolescent Mental Health Unit, Torrecárdenas University Hospital, Almería, Spain

**Keywords:** Attention-deficit/hyperactivity disorder, Dimensional approach, Virtual reality, CPT, Cluster analysis

## Abstract

In the past two decades, the traditional nosology of attention-deficit/hyperactivity disorder (ADHD) has been criticized for having insufficient discriminant validity. In line with current trends, in the present study, we combined a data-driven approach with the advantages of virtual reality aiming to identify novel behavioral profiles of ADHD based on ecological and performance-based measures of inattention, impulsivity, and hyperactivity. One hundred and ten Spanish-speaking participants (6–16 years) with ADHD (medication-naïve,* n* = 57) and typically developing participants (*n* = 53) completed AULA, a continuous performance test embedded in virtual reality. We performed hybrid hierarchical k-means clustering methods over the whole sample on the normalized t-scores of AULA main indices. A five-cluster structure was the most optimal solution. We did not replicate ADHD subtypes. Instead, we identified two clusters sharing clinical scores on attention indices, susceptibility to distraction, and head motor activity, but with opposing scores on mean reaction time and commission errors; two clusters with good performance; and one cluster with average scores but increased response variability and slow RT. DSM-5 subtypes cut across cluster profiles. Our results suggest that latency of response and response inhibition could serve to distinguish among ADHD subpopulations and guide neuropsychological interventions. Motor activity, in contrast, seems to be a common feature among ADHD subgroups. This study highlights the poor feasibility of categorical systems to parse ADHD heterogeneity and the added value of data-driven approaches and VR-based assessments to obtain an accurate characterization of cognitive functioning in individuals with and without ADHD.

**Supplementary Information:**

The online version contains supplementary material available at 10.1007/s00787-023-02250-4.

## Introduction

The diagnostic and statistical manual of mental disorders (DSM) has traditionally conceptualized the diagnosis of attention-deficit/hyperactivity disorder (ADHD) as consisting of two symptom domains of inattention and hyperactivity/impulsivity. Based on six out of nine criteria cut-offs, DSM delimits three ADHD subgroups: predominantly inattentive (ADHD-IA), predominantly hyperactive-impulsive (ADHD-HI), and combined (ADHD-C) presentations. Nonetheless, in the past two decades, the diagnostic validity of this taxonomy has been strongly criticized for having insufficient discriminant validity [1, 2]. Several studies have found similar neuropsychological profiles between ADHD-C and ADHD-IA subtypes [3–7]. These subtypes seem also not to differ from the ADHD-HI subtype on the level of inattention or functional outcomes [8, 9]. Besides, using DSM criteria, ADHD subtypes present substantial variability in symptom manifestation, clinical course, and treatment response [1, 2]. Such variability is attributable to the categorical nature of DSM diagnosis [10]. The use of nominal criteria for diagnosis means that restrictive and subthreshold symptom profiles can coexist within the same diagnostic label. Thus, behind a diagnosis of ADHD-IA, it is possible to find patients not only with a restrictive inattentive profile but also those with subthreshold symptoms of hyperactivity/impulsivity (≤ 5 criteria).

Dimensional approaches to psychopathology, such as the Research Domain Criteria [11] and the Hierarchical Taxonomy of Psychopathology [12], emerged in recent years aiming to disentangle symptom heterogeneity and create a reliable and clinically useful nosology for mental health. These initiatives propose a data-driven alternative to DSM and conceptualize psychopathological problems as a spectrum rather than categories with strict boundaries to “non-normality”. In this sense, the fifth edition of DSM [13] also tried to adopt a dimensional model, for example, by shifting to ADHD “presentations” from “subtypes” to recognize that symptomatology is not necessarily stable across development, or by including grades of ADHD severity. However, these modifications have not been enough to address the existing limitations and the scientific and clinical communities still appeal for a revised ADHD nosology [14]. On this matter, data-driven approaches are being increasingly encouraged to clarify within-diagnosis heterogeneity in ADHD. Previous research has identified novel ADHD subgroups using parent reports of temperament traits or performance-based measures of executive functioning (for a detailed review see [15]). None of these studies have obtained ADHD profiles congruent with DSM nosology. However, it should be noted that they have not attempted to define ADHD profiles using performance-based measures of the core symptom domains on which the current ADHD nosology is based. The incorporation of quantitative measures of inattention, impulsivity and hyperactivity to this field might be useful for understanding the validity problems of ADHD subtypes, as the current organization of symptoms is grounded in parents and teacher reports [2, 16, 17]. This approach might help to guide ADHD nosology reframing, as well as to address other important research concerns, such as the debate on whether a purely (“restricted”) inattentive ADHD subtype exists and to what extent should it be considered a distinct attention disorder [18–20].

Continuous Performance Tests (CPTs) are among the most popular paradigms to assess attentional impairments and impulsivity during sustained attention tasks [21]. In these tasks, children have to detect infrequent target stimuli among a sequence of non-target stimuli for an extended course of time. Standard variables of CPT performance include omission errors, commission errors, mean reaction time (RT), and Standard Deviation of RT (SDRT). Although CPTs have proved to be useful to complement the clinical diagnosis of ADHD [22], and to monitor the effects of pharmacological interventions [23–25], they have been criticized for having poor ecological validity and low sensitivity and specificity rates [26]. In recent years, these limitations have been overcome by the incorporation of virtual reality (VR) technology. According to Kessels (2019) [27], ecological validity refers, on the one hand, to the ability of a test to demand the same cognitive resources of everyday activities, in other words, to recreate the context in which impairments appear spontaneously. On the other hand, it is also understood as the ability of a test to predict the examinee’s functional abilities in daily life activities, even if it does not resemble everyday situations. Although, indeed, a CPT is not a common task in an academic context, it demands the attentional resources necessary to resemble school tasks. Thus, one of the contributions of VR has been to embed the CPTs in virtual classrooms, as academic settings are the usual scenario in which concentration problems are referred to by teachers and parents [21, 28]. Besides, VR technology has enabled the incorporation and quantification of two domains of interest for ADHD: motor activity and attentional distraction. Hyperactivity is a representative ADHD trait [13] but conventionally measured using subjective informant ratings given the lack of well-established and standardized objective methods. Using motion sensors incorporated in the glasses, Parsons et al. [29] reported increased head turnings during task performance in ADHD children, especially in the distractors condition. Real-world distracters (e.g., paper airplane flying, whispers, a car passing) during task performance have been demonstrated to negatively impact CPT performance in ADHD children in comparison to unaffected peers, in terms of increased rates of omission errors, commission errors, slow RT, and increased SDRT [29–32]. Perhaps the most innovative contribution in this field has come through the virtual CPT AULA (“classroom” in English), the only validated virtual CPT for children from 6 to 16 years [33]. AULA tracks head movements in relation to task stimuli so the test is not limited to reporting the level of motor activity, it informs about how much time children spent looking at environmental distractors (external distractions) and the number of errors they commit when the attentional focus is well-directed to target stimuli (internal distractions). This is a relevant aspect for the study of attentional lapses in ADHD since the tendency to get distracted with self-generated thoughts [34, 35] has been uniquely addressed through rating scales or thought probes [35, 36]. Considering the highlighted advantages, substantial evidence shows CPTs embedded in virtual reality provide increased ecological validity, a more accurate characterization of ADHD performance, and a greater ability to discriminate between children with and without ADHD [21, 32, 37].

Taken together, in the present study we unified current trends in data-driven driven approaches with the advantages of virtual CTPs. We aimed to identify novel behavioral profiles of ADHD based on ecological and performance-based measures of inattention, impulsivity, and hyperactivity, through the application of exploratory clustering analyses to the main outcome measures of the virtual CPT AULA. CPTs are commonly incorporated in neuropsychological assessment protocols for ADHD so we expected to find profiles that could serve as a guide for diagnosis and intervention planning. Based on prior subtyping studies, we expected between three to six subgroups of individuals with distinct attentional control profiles in ADHD and healthy-matched participants [38–41].

## Methods

### Procedure

Data were drawn from a database property of our research team which includes, from 2016 onwards, diagnostic information, questionnaires, IQ, and AULA performance on Spanish-speaking children and adolescents with neurodevelopmental disorders, mostly ADHD, and TD participants. This database contains data from routine clinical assessments at a neurorehabilitation center that includes the virtual CPT AULA in diagnostic assessment protocols. Families are invited to share the data for research purposes before the assessment. Children and adolescents referred to this clinic for possible ADHD undergo a neuropsychological assessment by a 10 years experienced child neuropsychologist [RC]. A parents’ semi-structured interview to gather medical and clinical information, behavioral ratings [Strengths and Difficulties Questionnaire (SDQ) [42]], Escalas Magallanes de Evaluación del Trastorno por Déficit de Atención con Hiperactividad (EMTDA-H) [43], Behaviour Rating Inventory of Executive Function (BRIEF) [44], and observations were used to determine DSM-5 criteria for ADHD. The database also includes data from a larger research project examining executive functions in ADHD. We recruited ADHD and TD children through mailing lists from public health and education services. Families willing to participate completed a phone interview to assess study eligibility. ADHD and TD participants underwent the same clinical assessment by a trained doctorate-level health psychologist [PFM]. Parents completed a clinical interview (The Kiddie Schedule for Affective Disorders and Schizophrenia (K-SADS-PL-5) [45] and a set of rating scales [SDQ [42], ADHD Rating Scale-5 (ADHD-RS-V) [46], Child Behavior Checklist (CBCL/6-18) [47], Conners 3 ADHD Index [48]] to determine DSM-5 criteria for ADHD. School interviews and reports were obtained whenever possible during both recruitment procedures.

All parents/legal guardians, and children over 12 years of age, provided verbal and written informed consent. Participants were assessed individually by an experienced psychologist [RC and PFM]. The virtual CPT was always administered first to avoid fatigue effects on attentional performance. Families received a brief assessment report. Ethical approval was obtained from local Institutional Ethics Committees.

For the goal of this study, we selected children with available scores on the SDQ, as it is the only shared ADHD rating scale across recruitment procedures.

### Participants

We selected children with a primary diagnosis of ADHD and TD controls. Two experienced psychologists rated ADHD diagnosis as ‘present’, ‘subthreshold’ or ‘absent’ following the abovementioned diagnostic procedures. As child ADHD symptoms are best conceptualized as a continuum [49, 50], we included children with subthreshold profiles in the ADHD group because they experience incapacitating symptoms (presence of 3–5 criteria) although they do not reach cut-off criteria [51]. TD participants must have no psychiatric history. Uncertain diagnoses were conferenced to consensus and excluded in case of disagreement. We excluded children from both groups if they have neurological illness, traumatic brain injury or genetic disorders; a diagnosis of intellectual disability, autism spectrum disorder, or psychosis; sensory or motor impairments that prevent completion of the task; IQ < 70; or any current or previous pharmacological treatment for ADHD symptoms, as long as medication improves CPT parameters at both computerized and virtual reality settings [23–25]. All participants were medication naïve because children had not been prescribed medication at the time of testing or child/parents’ objection to medication.

The final sample included 110 participants: 57 children with ADHD and 53 TD controls matched by age and IQ (Table 1). We did not have children with the ADHD-HI subtype due to its low prevalence, it is an improbable diagnosis after preschool that usually evolves into a combined presentation [2, 52]. The ADHD group scored significantly higher than the TD group on all the scales of the SDQ.

**Table 1:** Sample characteristics

Characteristic	ADHD (*n* = 57)	TD (*n* = 53)
Demographics		
Age, mean (SD)	9.47 (2.93)	10.34 (2.92)
Girls, *n* (%)	18 (31.58)^†^	32 (60.38)
IQ, mean (SD)^a^	102.19 (13.53)	108.81 (17.76)
European origin, *n* (%)	54 (94.74)	53 (100.00)
ADHD-Combined, *n* (%)	31 (54.38)	
ADHD-Inattentive, *n* (%)^b^	26 (45.62)	
Comorbid disorders, *n* (%)		
Specific learning disorder	10 (17.54)	
Language disorder	1 (1.75)	
Oppositional defiant disorder	1 (1.75)	
SDQ subscales–parents, mean (SD)		
Emotional symptoms	3.77 (2.33)^†^	2.55 (2.59)
Conduct problems	2.86 (2.18)^‡^	1.42 (1.61)
Inattention/hyperactivity	6.11 (2.19)^‡^	2.98 (2.25)
Peer problems	2.39 (2.31)	1.59 (1.61)
Prosocial behavior	7.98 (1.94)*	8.70 (1.69)
Total difficulties	15.07 (6.57)^‡^	8.34 (5.79)

Group-level comparisons were assessed via *t* tests and Chi-Square tests

^a^Participants recruited from the clinic were administered full-scale IQ as part of a recent diagnostic assessment while all other participants completed a short form (Vocabulary and Block Design) which correlates above 0.90 [53]

^b^Four participants had a subthreshold profile

^‡^*p* < 0.001

^†^*p* < 0.01

*p < 0.05

### Measures

#### Advanced virtual-reality test AULA

Using a head-mounted display (Samsung Gear VR), children are placed in a virtual classroom, sitting at a desk and looking at the blackboard. Children first perform a usability task (find and pop balloons) to get used to the 3D environment. They next complete two tasks (180 trials, 20% targets, each). First, they must press the button whenever they see on the blackboard or hear any stimulus other than the target (“*apple*”) (No-Go paradigm). Second, they are instructed to press the button whenever the target (“*seven*”) appears (Go paradigm). Target stimuli have a low probability of occurrence, so No-Go and Go tasks, respectively, generate conditions of over and under-stimulation intended to produce high-fast and low-slow response rates. During task performance, usual visual (e.g. student passing a note, raising the hand), auditory (e.g. whispers, car passing), and combined (e.g. pen drops, teacher’s walk) distracting stimuli from the school environment randomly appear (not interfering with items’ presentation) to increase ecological validity. Each paradigm is preceded by a practice run. Task specifications have been published elsewhere [21]. The complete administration lasts around 20 min.

AULA provides traditional measures of CPT paradigms such as: *Omissions*, missing responses to the target stimuli (as an index of inattention); *Standard deviation of Reaction Time *(*SDRT*) (a common index of response inconsistency [54]; *Mean hit RT* (often used as a measure of latency of response); and *Commissions*, responses to non-target stimuli (related to response inhibition). Besides, using the movement sensors placed in the virtual glasses, AULA registers how much and when the child moves the head, and how far the head deviates from the attentional focus (the blackboard, where visual target stimuli appear). This information is expressed in three novel variables: *Head motor activity*, defined as the sum of the averages of the three rotations (angles) of the X, Y and Z axes of the head and considered a quantitative measure of total head movements during the entire task; *Deviation of attentional focus*, defined as the amount of time in milliseconds that the child shifts the attention focus to any stimuli in the classroom other than the blackboard and interpreted as an index of external distractions (environmental stimuli) [30]; and *Quality of attentional focus*, defined as the total number of visual omission and commission errors that participants commit when the attentional focus is well-directed to the blackboard, and interpreted as attentional lapses due to internal distractors (thoughts).

AULA performance is quantified on normalized t-scores (ranging from 20 to 80) norm-referenced by age and sex groups [55]. T-scores ≤ 40 are interpreted as a very good performance; t-scores between 41 and 60, as average scores; t-scores between 61 and 70 (low performance), as a risk for attention problems; and t-scores between 70 and 80, as a high risk for attention problems (very low performance). AULA has reliability, specificity, and sensitivity rates above 90% [21] and an excellent convergent validity with goal standards such as the Conners’ CPT [56], the D2 test [57], and the Faces-Differences Perceptions Test [58].

#### Strengths and difficulties questionnaire (SDQ)-parents’ version

Parents completed the SDQ [42], an international and reliable scale to screen emotional and behavioral problems in children and adolescents aged 4–17 years. It contains 25 items divided between five scales: emotional symptoms, conduct problems, inattention/hyperactivity, peer relationship problems, and prosocial behavior.

### Statistical analysis

All analyses were run in R software [59].

#### Clustering approach

We applied hybrid hierarchical k-means clustering analyses to identify specific subgroups of attentional control among ADHD and TD participants. This algorithm first computed hierarchical clustering to select a tentative number of cluster centroids. We used Ward’s method (Euclidean distance) for agglomeration to minimize within-cluster variance in each iterative step. Then, cluster membership was determined through k-means analysis, starting the iteration process in the previously defined cluster centroids instead of in random seeds. This combination of clustering methods overcomes the limitations of each [60] and has been previously used by our group to identify novel phenotypes of compulsive behavior [61] and decision-making [62]. The algorithm was performed over the whole sample on the normalized t-scores of AULA main indices (*Omissions, SDRT, Deviation of attentional focus, Mean RT, Commissions,* and *Head movements*). We did not include the *Quality of attentional focus* because AULA provides this index for NoGo and Go paradigms separately instead of as a global index.

We examined several cluster solutions (*k*) ranging from 3 to 6 subgroups according to previous subtyping studies [38–41]. We inspected each cluster solution and decided on an appropriate cut-off guided by the majority rule of thirty clustering validation indices [63].

#### Group-level comparisons

Group-level differences were tested via robust models of ANOVA on 20% trimmed means and 2000 bootstrap samples for better control of Type I error [64, 65]. We performed one-way ANOVAs to compare performance in AULA’s main outcome measures according to DSM subtype and Cluster membership, as well as clusters’ demographics. Two-way mixed ANOVAs [66] were used to assess the effect of Group (DSM subtype/Cluster; between-subjects factor) and Task paradigm (No-Go vs Go task; within-subjects factor) on the variable *Quality of attentional focus*. All post-hoc tests applied Benjamini–Hochberg correction for multiple comparisons. Significance level was set at *p* < 0.05.

## Results

### DSM profiles of attentional control

**Fig. 1:**
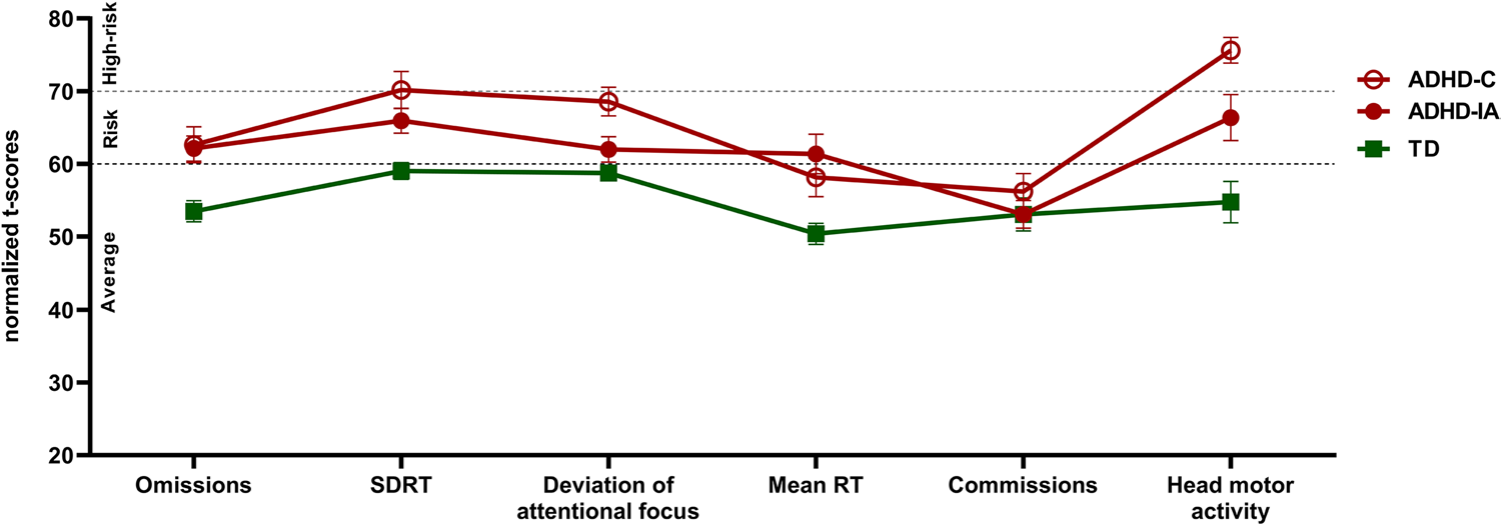
Attentional control profiles measured by the virtual CPT AULA of ADHD-Combined (ADHD-C), ADHD-Inattentive (ADHD-IA), and typically developing (TD) participants. 20% trimmed mean values of AULA main indices (t-scores): omission errors, standard deviation of reaction time (SDRT), time deviating the attentional focus from the blackboard, mean RT, commission errors, and total head movements. Error bars represent the 20% trimmed standard error of the mean. T-scores ≥ 61 represent a clinically low performance. Dashed lines indicate cut-offs for risk of attention problems (> 60 = at risk; > 70 = high risk)

ADHD-C and ADHD-IA subtypes showed a low-performing profile in AULA main indices (Fig. 1). Robust one-way ANOVA revealed significant effects for all outcome measures except for commission errors. Test statistics and mean differences for each post-hoc comparison are detailed in Supplementary Material (Table S1). Post-hoc tests adjusted for multiple comparisons showed that ADHD-C children obtained significantly worse scores than TD controls in all measures except for commission errors. Similarly, ADHD-IA children obtained significantly worse scores than TD controls in all measures except for commission errors and deviation from the attentional focus. When comparing subtypes, post-hoc tests revealed that ADHD-C children spent significantly more time deviating the attentional focus and performed significantly more head movements than ADHD-IA children.

Concerning the variable *Quality of attentional focus,* a robust two-way mixed ANOVA revealed main effects of DSM subtype [T_WJ(2, 35.45)_ = 4.23, *p* = 0.02] and task paradigm [T_WJ(1, 62.58)_ = 15.82, *p* < 0.001] but no interaction effect [T_WJ(2, 40.83)_ = 0.53, *p* = 0.57]. Concerning between-subjects effects, post-hoc tests corrected for multiple comparisons revealed that ADHD-C participants had a significantly lower performance than TD participants regardless of task paradigm (*p* = 0.02). We did not find significant differences between ADHD-C and ADHD-IA subtypes. Concerning within-subjects effects, scores on the *Quality of attentional focus* were significantly higher during the Go paradigm (*p* < 0.001). Mean values of each cluster per task paradigm are detailed in Table 2.

**Table 2:** Mean values of *Quality of attentional focus* according to DSM subtype

Task paradigm	ADHD-C	ADHD-IA	TD
NoGo task	57.63 (10.30)	56.75 (9.48)	52.67 (11.53)
Go task	**62.32 (8.64)**	59.69 (10.82)	55.27 (9.36)

20% trimmed mean values (normalized t-scores) and Standard Deviation are presented. Low scores (≥ 61) are boldfaced

### Data-driven profiles of attentional control

#### Best cluster solution

We graphically inspected cluster solutions ranging from 3 to 6 subgroups. For each *k* solution, we represented the performance profiles in AULA main indices and the percentage distribution of each cluster in ADHD and TD groups (Fig. S1). For *k* = 3, hybrid k-means analyses identified one low-performing subgroup constituted by 72.22% of ADHD participants; one subgroup with average scores (constituted by 80.65% TD participants); and one subgroup with intact performance but elevated SDRT and slow RT (formed by 52% TD participants). For *k* = 4, cluster analyses divided the low-performing cluster into two ADHD phenotypic subgroups, respectively, constituted by 89.92% and 48.19% ADHD participants. For *k* = 5, the cluster with average scores was split into average and high-performance subgroups. Finally, for a solution of *k* = 6, cluster analyses revealed one subgroup with high levels of head activity, a high tendency to deviate the attentional focus, and elevated SDRT.

According to the majority rule among thirty clustering validation indices, the five-cluster structure was the best cluster solution (Fig. S2) for explaining CPT performance among ADHD and TD participants. This structure demonstrated the highest internal consistency as stated by nine well-validated indices (Table S2). We also considered it a parsimonious solution to describe ADHD subpopulations.

#### Phenotypic characterization

**Fig. 2:**
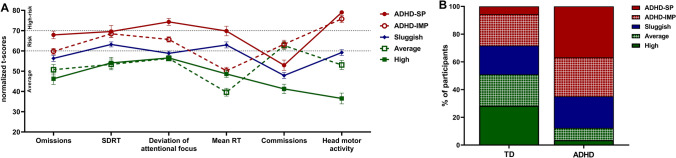
Attentional control profiles measured by the virtual CPT AULA according to the five-cluster solution. **A** 20% trimmed mean values of AULA main indices (t-scores): omission errors, standard deviation of reaction time (SDRT), time deviating the attentional focus from the blackboard, mean RT, commission errors, and total head movements. Error bars represent the 20% trimmed standard error of the mean. T-scores ≥ 61 represent a clinically low performance. Dashed lines indicate cut-offs for risk of attention problems (> 60 = at risk; > 70 = high risk). **B** Percentage distribution of each cluster in ADHD and TD groups

Figure 2A depicts the scores of the five clusters obtained in AULA’s main indices. Clusters were labeled according to their performance, following the clinical cut-off points provided by the validation study. We observed two low-performing subgroups with an opposite performance profile in latency of response and response inhibition. These clusters were, respectively, labeled ADHD-Slow Processing (ADHD-SP; *n* = 24; 87.5% ADHD participants) and ADHD-Impulsive (ADHD-IMP; *n* = 28; 57.14% ADHD participants). The clusters showing average scores were labeled *Average* (*n* = 17; 70.58% TD participants) and *High performers* (*n* = 17, 88.24% TD participants) as the latter group had better scores in omissions, commissions, and head activity. Finally, the fifth cluster was labeled *Sluggish* (*n* = 24; 54.16% ADHD participants) as it showed a relatively average performance in all variables but slightly clinically elevated scores in mean RT and SDRT. Figure 2B illustrates the percentage distribution of participants from each cluster in ADHD and TD groups. 64.91% of the ADHD sample belonged to ADHD-SP and ADHD-IMP clusters, while 28.81% belonged to the Sluggish cluster. 50.94% of TD participants were found in the *Average* and *High performing* clusters. 20.76% of TD participants belonged to the *Sluggish* cluster.

**Fig. 3:**
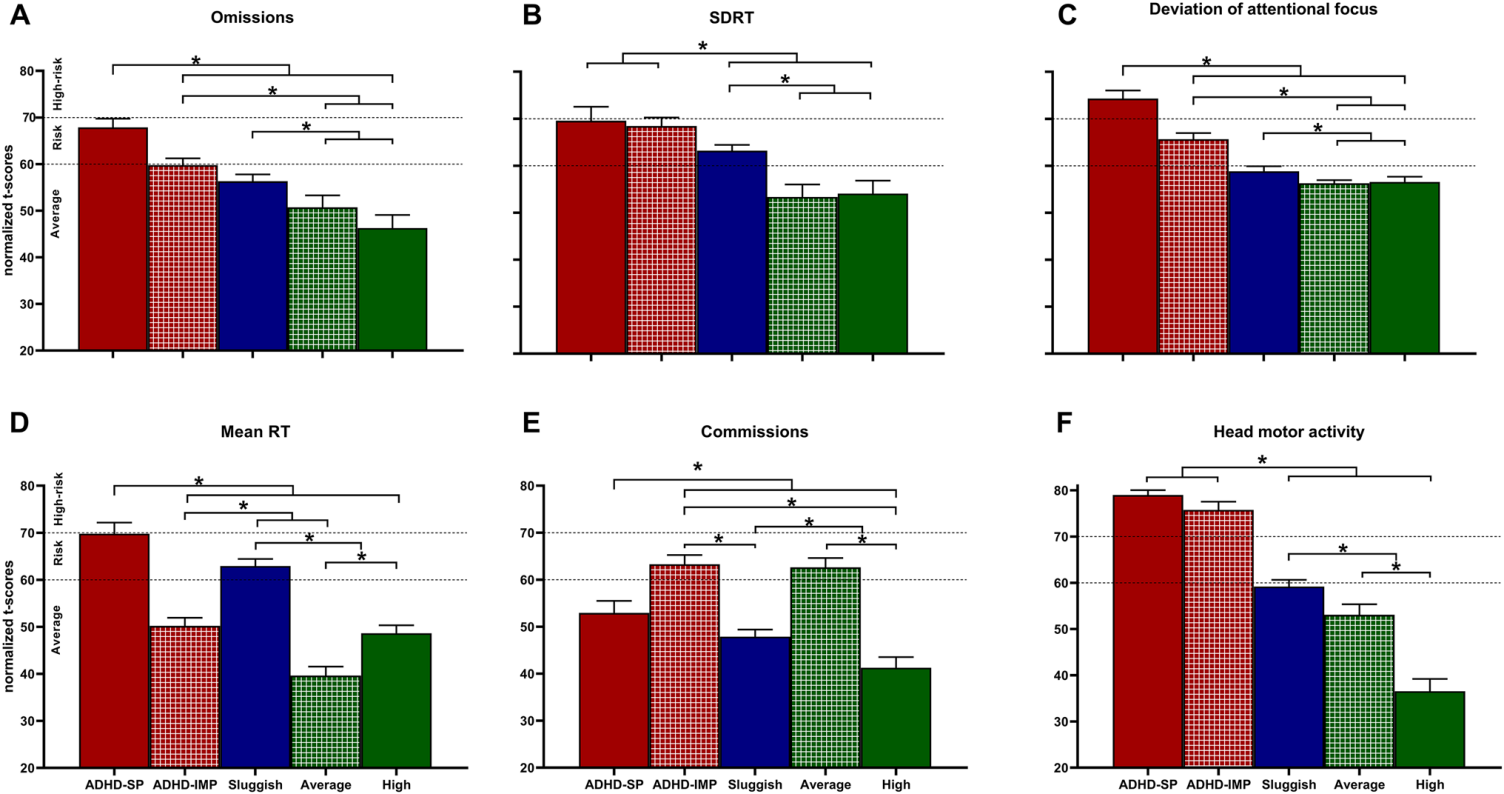
Post-hoc comparisons between the obtained clusters in the main indices of the virtual CPT AULA. 20% trimmed mean values of AULA main indices (t-scores) are presented. Error bars represent the 20% trimmed standard error of the mean. T-scores ≥ 61 represent a clinically low performance. Dashed lines indicate cut-offs for risk of attention problems (> 60 = at risk; > 70 = high risk). *Significant differences after adjustment for multiple comparisons using Benjamini–Hochberg correction

Robust one-way ANOVA showed statistically significant differences in all AULA outcome measures among clusters, yielding large effect sizes. Test statistics and mean differences for each comparison are included in the Supplementary Material (Table S3). Significant post-hoc comparisons after adjusting for multiple comparisons are represented in Fig. 3. Briefly, ADHD-SP and ADHD-IMP clusters significantly differed in omissions, mean RT, commission errors, and deviation from the attentional focus. Both clusters differed from average and high performers in most outcome measures. The Sluggish cluster presented an intermediate profile, as this cluster scored significantly worse than average and high performers but significantly better than ADHD-SP and ADHD-IMP clusters.

In the *Quality of attentional focus*, the ADHD-SP cluster obtained a clinically low score in both NoGo and Go paradigms, while the ADHD-IMP cluster only reached a clinically low performance in the Go task. Mean values for each cluster are detailed in Table 3. A robust two-way mixed ANOVA revealed significant main effects of task paradigm [T_WJ(1, 53.14)_ = 22.42, *p* < 0.001] and cluster profile [T_WJ(4, 31.04)_ = 18.93, *p* < 0.001], as well as a significant Task × Cluster interaction effect [T_WJ(4, 30.73)_ = 30.73, *p* = 0.02]. Concerning task paradigm, post-hoc tests adjusted for multiple comparisons revealed no significant differences between ADHD-SP and ADHD-IMP clusters neither in the Go nor in the NoGo task. In the No-Go task, high performers showed a significantly better score than the other clusters. ADHD-SP participants obtained a significantly worse performance than average performers. In the Go task, ADHD-SP and ADHD-IMP clusters obtained a significantly worse performance than average performers. The ADHD-SP also showed a significant worse score than Sluggish performers. In this task, high performers obtained a significantly better performance than ADHD-SP, ADHD-IMP and Sluggish participants. Regarding within-subjects effects, post-hoc analyses showed that ADHD-IMP and High performers obtained a significantly worse score in the Go task in comparison with the NoGo task. No differences in task paradigm were revealed for ADHD-SP, Sluggish and Average performers.

**Table 3:** Mean values of *quality of attentional focus* according to cluster membership

Task paradigm	ADHD-SP	ADHD-IMP	Sluggish	Average	High
NoGo task	**61.44 (8.23)**	56.67 (5.89)	54.56 (10.07)	53.36 (7.64)	40.36 (7.79)
Go task	**63.50 (6.73)**	**64.00 (8.58)**	55.06 (8.16)	55.09 (8.71)	49.64 (5.07)

20% trimmed mean values (normalized t-scores) and Standard Deviation are presented. Low scores (≥ 61) are boldfaced

#### Clusters’ characteristics

Clusters did not differ in IQ but did in age (*F*_*t*_ = 6.69, *p* = 0.01) and sex distribution (*p* = 0.01) (Table 4). We found that DSM-5 subtypes for ADHD were similarly distributed across cluster profiles. ADHD-SP and ADHD-IMP clusters obtained significantly higher scores in the Inattention/Hyperactivity subscale (*F*_*t*_ = 6.24, *p* = 0.003) and the Total difficulties score (*F*_*t*_ = 3.72, *p* = 0.03) of the SDQ.

**Table 4:** Clusters’ demographic characteristics

Characteristic	ADHD-SP (1)	ADHD-IMP (2)	Sluggish (3)	Average (4)	High (5)	Significant comparisons^b^
Demographics						
*n*	24	28	24	17	17	
Age, mean (SD)	9.25 (2.61)	8.25 (1.88)	10.08 (3.09)	10.71 (2.69)	12.41 (3.08)	5 > 1^†^, 3^*^ 2 < 3^*^, 4^*^, 5^‡^
Girls, *n* (%)	9 (37.50)	8 (28.57)	9 (37.50)	12 (70.59)	12 (70.59)	*p* = 0.01
IQ, mean (SD)	98.96 (12.69)	105.04 (16.08)	105.58 (15.07)	108.94 (19.55)	111.18 (15.91)	
European origin, *n* (%)	23 (95.83)	26 (92.86)	21 (87.05)	17 (100.00)	17 (100.00)	
Typically developing, *n* (%)	3 (12.50)	12 (42.86)	11 (45.84)	12 (70.58)	15 (88.24)	
ADHD-combined, *n* (%)	14 (58.33)	12 (42.86)	2 (8.33)	2 (11.77)	1 (5.88)	
ADHD-inattentive, *n* (%)^a^	7 (29.17)	4 (14.28)	11 (45.83)	3 (17.65)	1 (5.88)	
Comorbid disorders, *n* (%)						
Specific learning disorder	5 (20.83)	1 (3.57)	2 (8.33)	2 (11.77)	0 (0.00)	
Language disorder	0 (0.00)	1 (3.57)	0 (0.00)	0 (0.00)	0 (0.00)	
Oppositional defiant disorder	0 (0.00)	1 (3.57)	0 (0.00)	0 (0.00)	0 (0.00)	
SDQ subscales-parents, mean (SD)						
Emotional symptoms	4.08 (2.36)	2.96 (2.33)	3.00 (2.45)	2.12 (2.29)	3.59 (3.10)	
Conduct problems	3.00 (2.45)	2.79 (2.22)	1.75 (1.51)	1.29 (1.57)	1.41 (1.58)	
Inattention/hyperactivity	6.33 (1.50)	5.00 (2.57)	4.50 (2.62)	2.94 (2.70)	3.29 (2.59)	1 > 3^*^, 4^‡^, 5^‡^ 2 > 4^*^, 5^*^
Peer problems	2.92 (2.28)	1.82 (1.88)	1.79 (2.04)	1.18 (1.81)	2.12 (1.83)	
Prosocial behavior	8.13 (1.68)	8.29 (1.65)	8.29 (1.68)	8.65 (2.03)	8.41 (2.53)	
Total difficulties	16.29 (6.36)	12.50 (6.27)	11.04 (6.56)	7.53 (7.08)	9.82 (6.77)	1 > 3^†^, 4^‡^, 5^*^ 2 > 4^*^

20% trimmed means are presented

^a^Four participants had a subthreshold profile

^b^Significant effects after adjustment for multiple comparisons using Benjamini–Hochberg correction

^‡^*p* < 0.001

^†^*p* < 0.01

*p < 0.05

## Discussion

In the present study, we used the virtual CPT AULA to obtain an objective and ecological assessment of attentional control, impulsivity, and hyperactivity in a sample of 57 medication-naïve ADHD children and 57 TD controls. First, we compared the performance of ADHD-C and ADHD-IA subtypes to test the discriminant validity of DSM-5 criteria. We found that both subtypes showed t-scores above the clinical cut-off (> 60) in most AULA outcome measures, and significantly differed from TD controls. However, they showed an indistinguishable performance profile. We did not observe meaningful differences in variables that are theoretically supposed to discriminate between them, such as motor activity (as an index of hyperactivity) or commission errors (as an index of response disinhibition). This data might support the idea that DSM-5 criteria are useful for detecting ADHD individuals with functional impairments, but the taxonomy is not sensitive enough to discriminate among ADHD-C and ADHD-IA subtypes [2, 67]. Then, we proceeded to identify novel behavioral profiles of ADHD using clustering analyses on the main outcomes of the virtual CPT AULA. We found that ADHD and TD children were regrouped into five clusters that cut across DSM subtypes.

Most ADHD children belonged to two clusters with AULA scores above the clinical cut-off (t-score > 60). These clusters, ADHD-SP and ADHD-IMP, were characterized by elevated scores in omission errors, increased SDRT, and a high tendency to spend time distracted by external stimuli (*Deviation from the attentional focus*). These results support extensive literature on intra-individual variability in RT as a common feature among ADHD subtypes [54, 68, 69], as well as the negative impact of external distracting stimuli on attention performance [32, 70–72]. Moreover, ADHD-SP and ADHD-IMP clusters also showed clinically high levels of head motor activity. This result could have notable implications for ADHD taxonomy as approximately half of our ADHD sample belonged to the inattentive subtype. It is remarkable that we found high rates of head motor activity, above the clinical cut-off, in both ADHD-C and ADHD-IA subtypes, as well as in the two ADHD phenotypic clusters. We might suggest that children with ADHD-IA can display increased head motor activity during challenging tasks although they do not reach the cut-off criteria for impulsivity/hyperactivity symptoms. This finding is in agreement with the conceptualization of hyperactivity as a non-ubiquitous behavior triggered by highly cognitively demanding activities, such as CPTs [73–76]. In addition, this would also be in line with the idea that a restrictive inattentive ADHD subtype might not exist [18–20]. These findings might explain why previous studies have reported no differences in quantifiable measures (e.g., actigraphs) of gross motor activity between categorical ADHD subtypes [75, 77–79]. Parents’ reports of hyperactivity symptoms may not be consistent with the quantitative information obtained by objective motion measurements [22] so our findings support the valuable and complementary information that objective movement might add to clinical diagnosis.

ADHD-SP and ADHD-IMP clusters were only distinguishable by the latency of response (mean hit RT) and response inhibition (commission errors). While the ADHD-SP cluster was characterized by a clinically significant slow RT and an adequate rate of commission errors, the ADHD-IMP cluster showed adequate mean RT but elevated commissions. This opposing performance profile might suggest that latency of response and response inhibition might be useful domains to distinguish between ADHD subpopulations. It might be congruent with previous subtyping findings dissociating processing speed and interference control in ADHD [38, 39, 80]. Concerning internal distractions (*Quality of attentional focus*), briefly, we observed that ADHD-SP participants had significantly more attentional lapses (in terms of visual omissions and commission errors) having the attentional focus well-directed to target stimuli. This group obtained clinically low scores regardless of NoGo and Go paradigms. The ADHD IMP cluster, in contrast, just reach a clinically low performance in this domain in the Go task. We might hypothesize that children with an ADHD-SP profile, in which a slow latency of response is prominent, present aggravated attentional impairments during CPT performance, in terms of scores above the clinically high-risk cut-off (t-score > 70), and are more susceptible to both external and internal distractors. We might suggest a greater implication of mind-wandering experiences or sluggish cognitive tempo features in this subgroup [34, 81]. Those children with an ADHD-IMP profile, however, seem to be prone to get distracted by internal stimuli only in monotonous and low-response rate (vigilance) tasks. Further studies should employ direct measures of internal distractibility to explore the contribution of internal stimuli to attentional impairments in children with ADHD and its potential relationship with sluggish cognitive tempo or motivational processes.

The identification of two clusters with good performance, mainly constituted by TD children, allowed us to interpret the clinical significance of the above-mentioned ADHD profiles. ADHD-SP and ADHD-IMP children significantly differed from clusters with good performance. Besides, the performance profile of the ADHD-SP cluster closely resembled that of Sluggish and high-performing subgroups, in the same manner that the ADHD-IMP cluster mirrored average performers. We observed this parallelism between clinical and non-clinical clusters in all outcome measures of the virtual CPT AULA except for motor activity. As ADHD-SP and ADHD-IMP clusters are the only ones struggling with task performance, we might suggest that they experience a clinically significant increase in head movements to meet task demands [74]. These results may reinforce the dimensional character of ADHD [49, 50, 82], and suggest that behavioral variability during CPT performance might be similarly distributed in individuals with and without ADHD.

Finally, using the SDQ to externally validate our five clusters, we found that ADHD-SP and ADHD-IMP participants had higher impairment scores in the inattention/hyperactivity and total difficulties scales of the SDQ questionnaire. However, we did not observe significant differences in emotional, conduct, and peer problems. We should note that there are scales more adequate than the SDQ, such as the CBCL/6-18 [47], to perform a more exhaustive examination of internalizing and externalizing behaviors. However, previous clustering studies have reported no differences between clusters in ADHD and depressive symptoms [83], or externalizing, social, and academic problems [38]. As such, AULA performance does not entirely correlate to parents’ ratings in the ADHD Rating Scale-IV [84]. This could corroborate the assumption that performance-based measures and rating scales address different but complementary information [85]. Neuropsychological measures of executive functions seem to be weakly associated with subjective ratings of inattention, impulsivity, and hyperactivity [86, 87]. Questionnaires such as the SDQ might be useful to identify individuals with ADHD symptoms but are not specific enough to detect specific behavioral patterns. These findings highlight the necessity to move towards multi-source assessment methods including direct measures to improve the accuracy of ADHD diagnosis [88].

Altogether, our study highlights the poor feasibility of traditional categorical systems to parse ADHD heterogeneity and the added value of data-driven approaches and VR-based neuropsychological assessment to obtain an objective and less biased characterization of cognitive functioning in individuals with and without ADHD. Virtual CPTs, such as AULA, allow obtaining an integrative and ecological perspective of attentional control and, thus, understanding the internalizing and/or externalizing mechanisms underlying attentional impairments, which is critical to broadening our understanding of ADHD behavior. We identified two behavioral profiles of ADHD that could be mainly differentiated by the latency of response and response inhibition. Head motor activity, in contrast, seems to be a common feature among ADHD subgroups. These objective and ecological parameters could serve to refine diagnostic criteria for ADHD subtypes and help clinicians in planning personalized interventions.

Our results replicate a previous clustering study focused on testing the external validity of AULA in the Spanish population [89]. Unpublished data from our research group have also replicated these cluster profiles in larger samples including medicated and medication-naïve children with ADHD and TD in the Latin population. Nonetheless, some limitations in this study should be noted. The sample is not fully representative as we could have neither children with the ADHD-HI presentation nor children with subthreshold diagnoses of ADHD-C and ADHD-HI. Moreover, we included a small number of children with subthreshold ADHD-IA. Although we performed cluster analyses to a similar sample size to previous studies [38, 62, 83], our findings should be replicated in larger samples addressing these issues. Further studies should also examine other measures of internalizing and externalizing symptoms and collect functional outcomes to externally validate the clusters.

### Supplementary Information

Below is the link to the electronic supplementary material.

Supplementary file1 (DOCX 3980 KB)

## Data Availability

The data and source code that support the findings of this study are openly available in Open Science Framework at osf.io/j2rzb.
