# Supplementary material for: Data-driven profiles of attention-deficit/hyperactivity disorder using objective and ecological measures of attention, distractibility, and hyperactivity
Source: Eur Child Adolesc Psychiatry. 2023 Jun 30;33(5):1451–63. doi: 10.1007/s00787-023-02250-4 (PMC11098896; doi:10.1007/s00787-023-02250-4)
Supplement: Supplementary file 1 — Supplementary file1 (DOCX 3980 KB) [file 787_2023_2250_MOESM1_ESM.docx]

*Supplementary material*

Data-Driven Profiles of Attention-Deficit/Hyperactivity Disorder Using Objective and Ecological Measures of Attention, Distractibility and Hyperactivity.

Pilar Fernández-Martín[
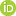
](https://orcid.org/0000-0002-0792-3276)^1,2^, Rocío Rodríguez-Herrera[
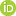
](https://orcid.org/0000-0002-2367-2091)^1, 2^, Rosa Cánovas López[
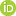
](https://orcid.org/0000-0001-9227-5893)^3^, Unai Díaz-Orueta[
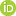
](https://orcid.org/0000-0002-0349-8890)^4,5^, Alma Martínez de Salazar Arboleas[
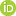
](https://orcid.org/0000-0002-4506-2817)^6^, and Pilar Flores Cubos[
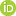
](https://orcid.org/0000-0001-7950-2086)^1, 2, 3^

^1^ Department of Psychology, Faculty of Psychology, University of Almeria, Spain

^2^ Health Research Center (CEINSA), University of Almeria, Almería, Spain

^3^ Neurorehabilitation and Autonomy Center Imparables, Almería, Spain

^4^ Department of Psychology, Maynooth University, Ireland

^5^ International University of la Rioja (UNIR), Spain

^6^ Child and Adolescent Mental Health Unit, Torrecardenas University Hospital, Almería, Spain

[**Table S1. Robust One-way ANOVA in AULA’s main outcome measures according to DSM subtype.** 3](#_Toc133323794)

[**Table S2. Clustering validation indices for *k* solutions ranging from 3 to 6 subgroups.** 4](#_Toc133323795)

[**Table S3. Robust One-way ANOVA in AULA’s main outcome measures according to the five-cluster solution.** 5](#_Toc133323796)

[**Fig. S1. Attentional control profiles measured by the virtual CPT AULA according to k cluster solutions.** 6](#_Toc133323797)

[**Fig. S2. Selection of best cluster solution among thirty validation indices** 7](#_Toc133323798)

[**References** 8](#_Toc133323799)

| **Robust One-way ANOVA** | **Omissions** | **SDRT** | **Deviation of attentional focus** | **Mean RT** | **Commissions** | **Head movements** |
| --- | --- | --- | --- | --- | --- | --- |
| ***F_t_*** | 9.65^‡^ | 11.15^‡^ | 11.23^*^ | 8.09^†^ | 0.60 | 20.54^‡^ |
| **Effect size^a^** | 0.50 | 0.50 | 0.56 | 0.42 | 0.18 | 0.59 |
| **Post-hocs comparisons^b^** |  |  |  |  |  |  |
| ADHD-C vs TD | -9.15^†^  (-15.69, -2.68) | -11.13^†^  (-17.07, -3.46) | -9.82^‡^  (-14.89, -5.25) | -7.76^*^  (-14.87, -0.52) | -3.15  (-10.53, 4.69) | -20.87^‡^  (-28.20, -12.51) |
| ADHD-IA vs TD | -8.64^‡^  (-13.80, -3.25) | -6.91^†^  (-12.30, -1.52) | -3.24  (-9.51, 0.79) | -10.98^†^  (-18.38, -2.45) | -0.00  (-6.13, 6.98) | -11.62^†^  (-21.01, -2.05) |
| ADHD-C vs ADHD-IA | 0.51  (-6.30, 7.60) | 4.22  (-3.91, 11.29) | 6.58^*^  (0.09, 12.77) | -3.22  (-12.66, 6.53) | 3.15  (-3.97, 10.35) | 9.26^*^  (0.64, 16.69) |

**Table S1. Robust One-way ANOVA in AULA’s main outcome measures according to DSM subtype.**

^a^ The explanatory measure of effect size (ξ), a robust generalization of Pearson’s correlation allowing assumptions deviations and unequal sample sizes. Analogue to Cohen’s *d*, ξ = 0.15, 0.35 and 0.50 roughly correspond to small, medium and large effect sizes [1, 2].

^b^ 20% trimmed mean differences (t-scores) and associated bootstrap confidence interval are presented. Significant effects after adjustment for multiple comparisons using Benjamini-Hochberg correction.

^‡^*p*<.001, ^†^*p*<.01 and **p*<.05

| **Clustering index** | ***k* = 3** | ***k* = 4** | ***k* = 5** | ***k* = 6** |
| --- | --- | --- | --- | --- |
| *KL* | 0.22 | 0.96 | **9.22** | 0.389 |
| *CH* | **44.66** | 41.08 | 39.80 | 35.31 |
| *Hartigan* | 19.12 | 17.33 | **7.56** | 8.00 |
| *CCC* | **13.13** | 12.71 | 12.64 | 11.85 |
| *Scott* | 687.75 | 786.49 | **892.21** | 939.34 |
| *Marriot* | 1.32E+24 | 9.53E+23 | **5.70E+23** | 5.35E+23 |
| *TrCovW* | 83367366 | **54711169** | 41779104 | 39909689 |
| *TraceW* | 51984.21 | 44102.2 | **37905.34** | 35360.63 |
| *Friedman* | 413.097 | 466.3343 | **552.4537** | 589.86 |
| *Rubin* | 46.27 | 54.54 | **63.46** | 68.02 |
| *Cindex* | 0.37 | 0.33 | 0.31 | **0.30** |
| *DB* | **1.46** | 1.54 | 1.48 | 1.49 |
| *Silhouette* | 0.19 | 0.19 | **0.22** | 0.20 |
| *Duda* | **0.71** | 0.69 | 0.76 | 0.71 |
| *Pseudot2* | **22.72** | 13.72 | 9.46 | 9.65 |
| *Beale* | **1.53** | 1.65 | 1.17 | 1.49 |
| *Ratkowsky* | **0.37** | 0.35 | 0.33 | 0.31 |
| *Ball* | 17328.07 | **11025.55** | 7581.07 | 5893.44 |
| *Ptbiserial* | **0.49** | 0.43 | 0.46 | 0.43 |
| *Frey* | 0.98 | 0.09 | 0.88 | 0.55 |
| *McClain* | **1.02** | 1.89 | 2.24 | 2.80 |
| *Dunn* | 0.14 | 0.14 | **0.14** | 0.14 |
| *Hubert* | 0 | 0 | 0 | 0 |
| *SDindex* | 0.13 | 0.13 | **0.13** | 0.15 |
| *Dindex* | 20.49 | 18.82 | 17.39 | 16.7375 |
| *SDbw* | 0.85 | 0.61 | 0.40 | **0.37** |

**Table S2. Clustering validation indices for *k* solutions ranging from 3 to 6 subgroups.** The best cluster solution according to the majority rule among all thirty indices was a five-cluster structure. Best fitting values for each index are boldfaced. Indices were implemented from the NbClust package [3]. Each index computation and interpretation has been detailed elsewhere [3].

| **Robust One-way ANOVA** | **Omissions** | **SDRT** | **Deviation of attentional focus** | **Mean RT** | **Commissions** | **Head movements** |
| --- | --- | --- | --- | --- | --- | --- |
| ***F_t_*** | 15.28^‡^ | 10.00^‡^ | 34.98^‡^ | 41.67^‡^ | 23.63^‡^ | 96.06^‡^ |
| **Effect size^a^** | 0.77 | 0.73 | 0.94 | 0.89 | 0.74 | 0.83 |
| **Post-hoc comparisons^b^** |  |  |  |  |  |  |
| ADHD-SP vs ADHD-IMP | 8.10^‡^  (1.51, 14.69) | 1.12  (-8.37, 8.65) | 8.65^‡^  (1.98, 13.82) | 19.59^‡^  (11.66, 26.97) | -10.34^‡^  (-17.97, -3.01) | 3.22  (-1.69, 8.55) |
| ADHD-SP vs Sluggish | 11.56^‡^  (5.94, 18.00) | 6.38*  (-1.75, 13.63) | 15.50^‡^  (10.19, 20.25) | 6.88^†^  (-0.50, 13.63) | 5.06*  (-3.00, 14.44) | 19.81^‡^  (15.00, 24.50) |
| ADHD-SP vs Average | 17.14^‡^  (8.95, 26.26) | 16.20^‡^  (4.78, 26.61) | 18.04^‡^  (13.10, 23.14) | 30.18^‡^  (21.66, 38.05) | -9.70^†^  (-17.53, -0.31) | 25.91^‡^  (18.05, 33.86) |
| ADHD-SP vs High | 21.60^‡^  (10.82, 30.10) | 15.47^‡^  (6.06, 26.26) | 17.77^‡^  (12.91, 23.61) | 21.18^‡^  (13.94, 29.31) | 11.66^‡^  (3.71, 18.99) | 42.45^‡^  (34.51, 49.79) |
| ADHD-IMP vs Sluggish | 3.47  (-2.21, 9.33) | 5.26^†^  (0.17, 10.97) | 6.85^‡^  (3.25, 12.26) | -12.72^‡^  (-19.06, -7.58) | 15.40^‡^  (7.85, 21.43) | 16.59^‡^  (9.77, 22.42) |
| ADHD-IMP vs Average | 9.05^‡^  (0.92, 17.30) | 15.08^‡^  (7.65, 23.33) | 9.39^‡^  (5.80, 15.16) | 10.59^‡^  (3.99, 16.64) | 0.64  (-6.80, 9.40) | 22.69^‡^  (13.34, 31.93) |
| ADHD-IMP vs High | 13.51^‡^  (3.41, 21.54) | 14.35^‡^  (7.22, 24.31) | 9.12^‡^  (5.67, 15.38) | 1.59  (-5.25, 8.31) | 22.01^‡^  (14.40, 28.80) | 39.23^‡^  (30.58, 47.48) |
| Sluggish vs Average | 5.58*  (-1.99, 14.52) | 9.82^‡^  (2.47, 17.64) | 2.54*  (-0.82, 6.69) | 23.30^‡^  (16.94, 29.94) | -14.76^‡^  (-21.11, -5.83) | 6.10*  (-2.11, 13.45) |
| Sluggish vs High | 10.04^†^  (-0.19, 17.17) | 9.10^‡^  (2.18, 18.90) | 2.27  (-0.97, 6.88) | 14.30^‡^  (9.01, 20.83) | 6.60^†^  (-0.62, 13.82) | 22.64^‡^  (13.86, 31.21) |
| Average vs High | 4.45  (-7.09, 14.00) | -0.73  (-9.91, 11.27) | -0.27  (-3.27, 4.18) | -9.00^†^  (-15.00, -2.27) | 21.36^‡^  (11.91, 29.36) | 16.55^‡^  (6.45, 27.09) |

# **Table S3. Robust One-way ANOVA in AULA’s main outcome measures according to the five-cluster solution.**

^a^ The explanatory measure of effect size (ξ), a robust generalization of Pearson’s correlation allowing assumptions deviations and unequal sample sizes. Analogue to Cohen’s *d*, ξ = 0.15, 0.35 and 0.50 roughly correspond to small, medium and large effect sizes [1, 2, 4].

^b^ 20% trimmed mean differences (t-scores) and associated bootstrap confidence interval are presented. Significant effects after adjustment for multiple comparisons using Benjamini-Hochberg correction.

^‡^*p*<.001, ^†^*p*<.01 and **p*<.05


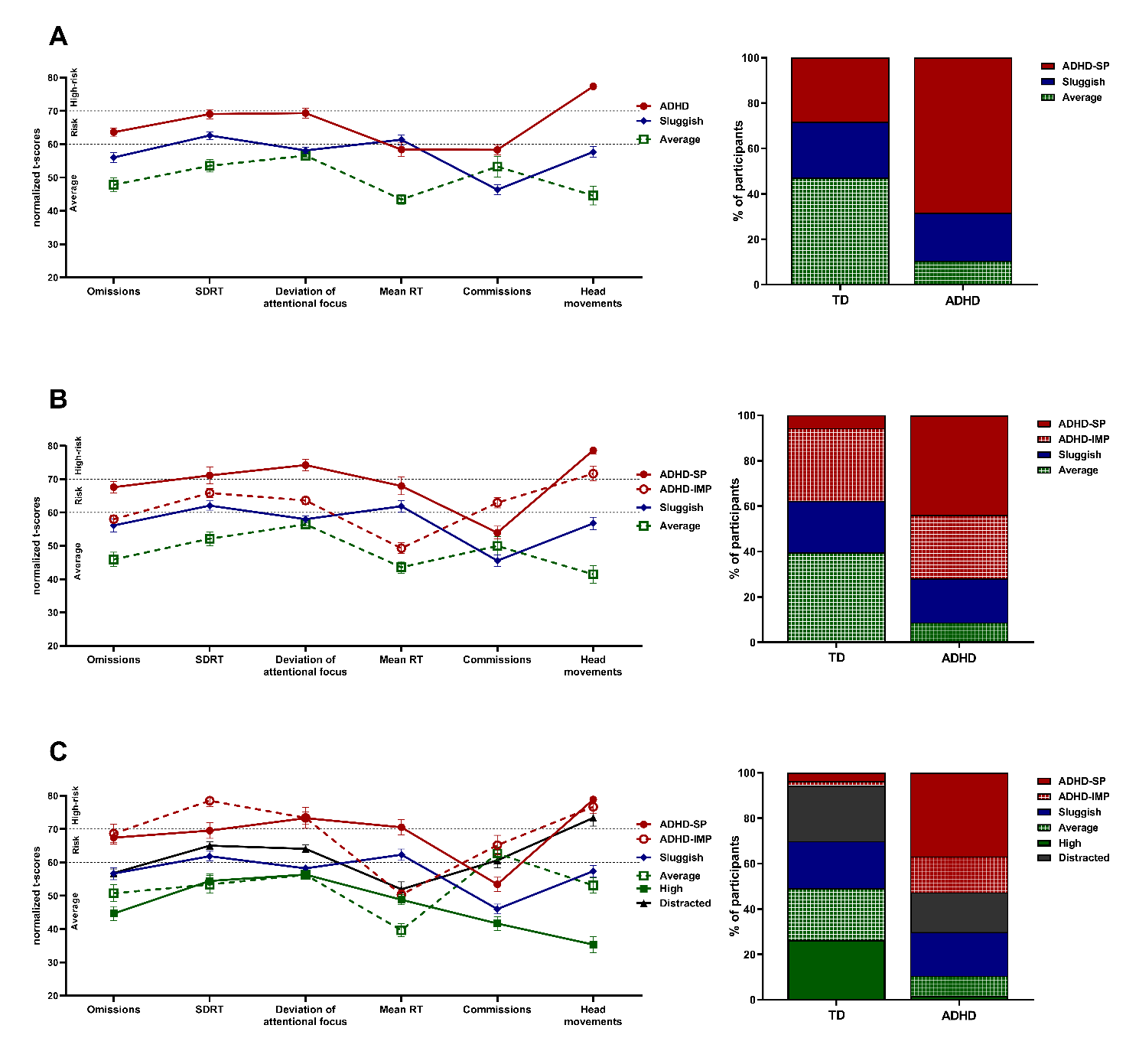


**Fig. S1. Attentional control profiles measured by the virtual CPT AULA according to k cluster solutions.** On the left, 20% trimmed mean values of AULA main indices (t-scores): omission errors, standard deviation of reaction time (SDRT), time deviating the attentional focus from the blackboard, mean RT, commission errors, and total head movements. Error bars represent the 20% trimmed standard error of the mean. T-scores ≥ 61 represent a clinically low performance. Dashed lines indicate cut-offs for risk of attention problems (>60 = at risk; >70 = high risk). On the right, percentage distribution of each cluster in ADHD and TD groups.

Graphical visualization for *k* = 5 is included in the manuscript as it is the best cluster structure to explain CPT performance among ADHD and TD children.


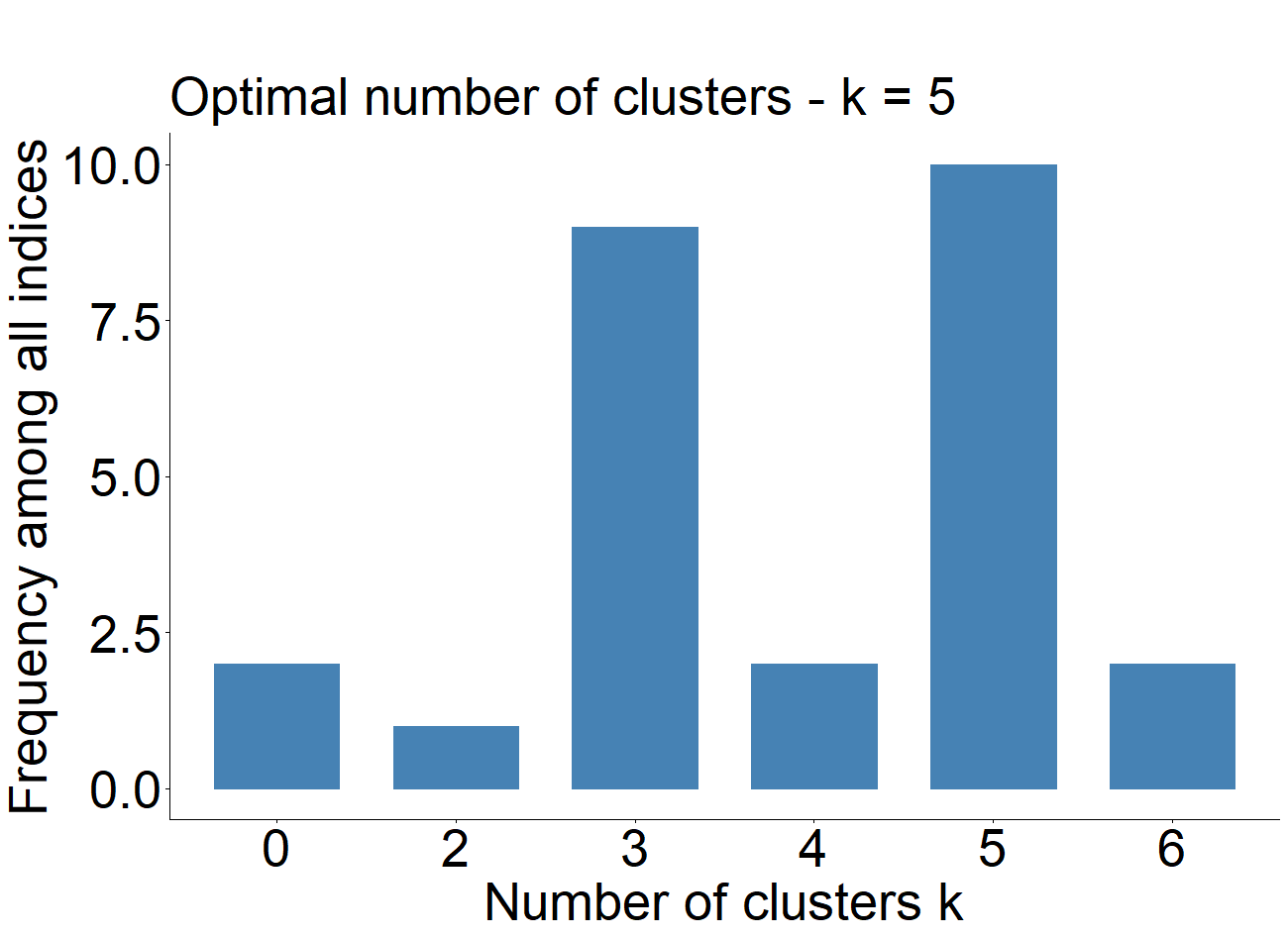


**Fig. S2. Selection of best cluster solution among thirty validation indices.** Ten clustering validation indices chose a five-cluster structure as the most optimal cluster solution.

# **References**

1. Wilcox RR, Tian TS (2011) Measuring effect size: A robust heteroscedastic approach for two or more groups. J Appl Stat 38:1359–1368. https://doi.org/10.1080/02664763.2010.498507

2. Wilcox R (2017) One-Way and Higher Designs for Independent Groups. In: Introduction to Robust Estimation and Hypothesis Testing: 4th Edition. Academic Press Inc., pp 319–415

3. Charrad M, Ghazzali N, Boiteau V, Niknafs A (2014) Nbclust: An R package for determining the relevant number of clusters in a data set. J Stat Softw 61:1–36. https://doi.org/10.18637/jss.v061.i06

4. Wilcox R (2017) Comparing Two Groups. In: Introduction to Robust Estimation and Hypothesis Testing: 4th Edition. Academic Press Inc., pp 145–234
